# The acute effect of high-dose supplemental oxygen on haemodynamics assessed by echocardiography in patients with pulmonary vascular disease living in Quito at 2850 m: a randomized, single-blind, placebo-controlled crossover trial

**DOI:** 10.1093/ehjopen/oeae097

**Published:** 2024-12-02

**Authors:** Julian Müller, Mona Lichtblau, Stéphanie Saxer, Mirjam Schmucki, Michael Furian, Simon R Schneider, Joël J Herzig, Meret Bauer, Diego Saragoni, Esther I Schwarz, Elizabeth Cajamarca, Rodrigo Hoyos, Silvia Ulrich

**Affiliations:** Department of Pulmonology, University Hospital Zurich, Rämistrasse 100, 8091 Zurich, Switzerland; Faculty of Medicine, University of Zurich, Pestalozzistrasse 3, 8032 Zurich, Switzerland; Department of Pulmonology, University Hospital Zurich, Rämistrasse 100, 8091 Zurich, Switzerland; Faculty of Medicine, University of Zurich, Pestalozzistrasse 3, 8032 Zurich, Switzerland; Department of Pulmonology, University Hospital Zurich, Rämistrasse 100, 8091 Zurich, Switzerland; Department of Health, Eastern Swiss University of Applied Sciences, Rosenbergstrasse 59, 9000 St. Gallen, Switzerland; Department of Pulmonology, University Hospital Zurich, Rämistrasse 100, 8091 Zurich, Switzerland; Faculty of Medicine, University of Zurich, Pestalozzistrasse 3, 8032 Zurich, Switzerland; Department of Pulmonology, University Hospital Zurich, Rämistrasse 100, 8091 Zurich, Switzerland; Department of Pulmonology, University Hospital Zurich, Rämistrasse 100, 8091 Zurich, Switzerland; Department of Pulmonology, University Hospital Zurich, Rämistrasse 100, 8091 Zurich, Switzerland; Department of Pulmonology, University Hospital Zurich, Rämistrasse 100, 8091 Zurich, Switzerland; Department of Pulmonology, University Hospital Zurich, Rämistrasse 100, 8091 Zurich, Switzerland; Faculty of Medicine, University of Zurich, Pestalozzistrasse 3, 8032 Zurich, Switzerland; Department of Pulmonology, University Hospital Zurich, Rämistrasse 100, 8091 Zurich, Switzerland; Faculty of Medicine, University of Zurich, Pestalozzistrasse 3, 8032 Zurich, Switzerland; Pneumology Unit, Carlos Andrade Marín Hospital, Av. Universitaria, 170103 Quito, Ecuador; Pneumology Unit, Carlos Andrade Marín Hospital, Av. Universitaria, 170103 Quito, Ecuador; Department of Pulmonology, University Hospital Zurich, Rämistrasse 100, 8091 Zurich, Switzerland; Faculty of Medicine, University of Zurich, Pestalozzistrasse 3, 8032 Zurich, Switzerland

**Keywords:** Pulmonary vascular disease, PVD, High altitude, Oxygen therapy, Pulmonary hypertension

## Abstract

**Aims:**

More than 220 Mio people live at altitudes above 2000 m, many of whom have pre-existing chronic diseases, including pulmonary vascular diseases (PVDs) such as pulmonary arterial hypertension (PAH) or chronic thromboembolic pulmonary hypertension (CTEPH). We investigated the acute effects of high-dose supplemental oxygen on pulmonary haemodynamics assessed by echocardiography in patients with PVD permanently living at 2850 m.

**Methods and results:**

In a randomized, single-blind, placebo-controlled crossover trial, patients with PVD diagnosed with PAH or CTEPH were allocated to receive 10 L/min supplemental oxygen (FiO_2_ ≈ 95%) and placebo air administered via a facial mask with reservoir near their living altitude in Quito at 2850 m (FiO_2_0.21, PiO_2_ ≈ 60% of sea level) in random order with a washout period of >2 h. After >15 min of breathing the respective FiO_2_, systolic pulmonary artery pressure (sPAP), cardiac output (CO), and other parameters were assessed by echocardiography. Furthermore, radial arterial blood gases were analysed. Twenty-eight patients with PVD (24 females, 26 PAH, age 45 ± 12 years) treated with phosphodiesterase-5 inhibitors (*n* = 28) and endothelin receptor antagonists (*n* = 9) were included. With oxygen vs. placebo air, sPAP was 57 ± 23 vs. 68 ± 24 mmHg, mean difference −11 mmHg (−15 to −6 mmHg, *P* < 0.001), CO was 3.2 ± 0.9 vs. 3.9 ± 1.1 L/min; −0.7 L/min (−0.9 to −0.4 L/min, *P* < 0.001), while sPAP/CO was unchanged, and the right ventriculo-arterial coupling was increased. PaO_2_ was 22.5 ± 9.7 vs. 7.6 ± 1.5 kPa; 14.9 kPa (11.4–18.4 kPa, *P* < 0.001).

**Conclusion:**

High-dose oxygen therapy in prevalent patients with PVD living near 2850 m significantly lowered sPAP but also CO by a reduced heart rate, resulting in an unchanged pulmonary resistance. Whether longer-term oxygen therapy would improve pulmonary vascular resistance requires further investigation.

**Registration:**

NCT06084559 URL: https://clinicaltrials.gov/study/NCT06084559.

## Introduction

In the absence of predominant lung disease, the two major forms of precapillary pulmonary hypertension (PH) are pulmonary arterial hypertension (PAH) and chronic thromboembolic PH (CTEPH), hereafter summarized as pulmonary vascular disease (PVD).^1^ PVD needs to be diagnosed by right heart catheterization (RHC). In Europe, where the majority of the population lives below 1000 m of altitude, PVD is rare with a reported prevalence that ranges from 26 to 55 cases per million adults.^2^ However, the systolic pulmonary arterial pressure (sPAP) increases with altitude, even in healthy individuals, due to hypoxic pulmonary vasoconstriction. Typically healthy people rarely have a sPAP >30 mmHg at 2850 m.^3^ Nevertheless, in subjects with elevated pulmonary arterial pressures living at high altitude, it is important to distinguish between isolated elevated PAP in healthy individuals at high altitude, highlanders with exaggerated PAP (high-altitude PH), and patients with true PVD exhibit pathophysiological changes (e.g. remodelling of the pulmonary vessels with plexiform lesions) identical to those in low-altitude inhabitants.^3–5^

However, the accurate diagnosis of PVD is difficult, even more in countries with limited medical infrastructure, resources, and is not possible without the access to RHC.^6^ Furthermore, to date, the diagnosis of PH is based on values assessed at low altitude.^1^ Many large cities, such as Quito (Ecuador) or Mexico City (Mexico), are located at high altitudes, and it is assumed that the worldwide population permanently living above 2000 m altitude encompasses >220 million people. With such an amount of people living at altitude, it is rather unsurprising to find among them symptomatic patients at living altitudes between 2000 and 3000 m who have severe precapillary PH with a clear PVD phenotype. Previous randomized controlled trials showed that supplemental oxygen therapy, applied in different settings (nocturnal, 5-week domiciliary, during exercise, RHC) improves haemodynamics, exercise capacity, symptoms, and quality of life in patients with PVD living <1000 m.^7–9^

Therefore, the aim of this study was to investigate the effect of high-dose oxygen therapy on pulmonary haemodynamics in patients with PVD diagnosed by RHC and classified as PAH or distal CTEPH, living in Quito near 2850 m of altitude.

## Methods

### Study design, randomization, and subjects

This was a randomized, single-blind, placebo-controlled crossover trial conducted between 24 September and 26 September at the Carlos Andrade Marín Hospital in Quito, Ecuador, which is located at 2850 m. Randomization to study sequences (oxygen–placebo/placebo–oxygen) was performed by software-based blocks with random computed block length and allocation concealment. Participants of all sexes with PAH or CTEPH diagnosed with RHC according to the haemodynamic criteria of recent guidelines were recruited from the Pulmonology Department in Quito between January 2023 and September 2023.^1^ Other forms of PH were excluded. Chronic thromboembolic pulmonary hypertension was diagnosed or excluded by pulmonary angiography. Participants were under stable conditions with unchanged PH-targeted medication for >4 weeks and gave their written informed consent. A resting SpO_2_ < 80% in Quito, having any inabilities to follow the study protocol, pregnancy, or breast feeding were exclusion criteria. The study complied with the Declaration of Helsinki, was approved by the local ethical authorities, and was registered on clinicaltrials.gov (NCT06084559).

### Intervention

According to the crossover design, patients received both interventions in a randomized order with a washout period of >2 h in between. The experimental intervention was calm breathing of 10 L/min supplemental oxygen (FiO_2_ ≈ 95%). The control intervention was calm breathing of placebo air (FiO_2_0.21, PiO_2_ ≈ 60% of sea level); both interventions were supplied via a facial mask with reservoir in supine or lateral position. The setting of both interventions was identical, and participants were blinded to the interventions. Patients who were treated with nocturnal oxygen therapy were asked to pause the therapy for the study.

### Assessments

Echocardiography was performed after 15 min of rest while breathing oxygen or placebo air, respectively. Echocardiographic recordings were conducted using a real-time sector scanner (CX 50, Philips, Philips Respironics, Zofingen, Switzerland) with an integrated colour, continuous-wave (CW), and pulsed-wave Doppler system. Recordings and measurements were performed according to guidelines of the American Society of Echocardiography.^10^ To estimate sPAP, the maximal pressure gradient of the tricuspid regurgitation was calculated from the maximal tricuspid regurgitation velocity (TRV) determined with CW Doppler using the modified Bernoulli equation: ΔPressure = 4 × TRV_max_^2^ with adding the right atrial pressure. Four experienced and well-trained investigators performed the echocardiographic imaging according to standard operating procedures. Repeated investigations of each patient with both conditions (air and oxygen therapy) were performed by the same investigator to minimize inter-observer variance. The analysis of the echocardiographic data was conducted by the same investigator who performed the imaging and was supervised by the principal investigator to ensure uniformity in measurements. Heart rate (HR) was derived continuously from a 3-lead ECG, and blood pressure was measured using an arm cuff. After the echocardiography while still breathing oxygen or placebo air, respectively, arterial blood samples were taken from the radial artery for immediate analysis of arterial blood gases (epoc Blood Analysis System, Siemens, Berlin, Germany). Baseline characteristics were collected from medical records from the Carlos Andrade Marín Hospital in Quito.

### Statistical analysis and sample size

Based on the primary outcome sPAP, a sample size of 24 was calculated, assuming an minimal clinically important difference of 5 mmHg, a standard deviation of 5 mmHg applying a significance level alpha of 0.05 and a power 0.9. To account for dropouts, we aimed to include 28 participants.

### Primary analysis

To compare the main outcome between oxygen and placebo air after 15 min of calm breathing, physiological data were summarized as means ± standard deviation. The distribution of the data was checked by visual inspection of histograms and Shapiro–Wilk test. Furthermore, test assumptions for linear mixed models such as homogeneity and normality of the residuals were checked visually with Tukey–Anscombe plots and *Q–Q* plots. A linear mixed model was fitted to the data using intervention (oxygen vs. placebo air), period, and intervention–period interaction as fixed effects and subject (id) as random intercept, so that carry-over effects (treatment-period interaction) and period effects were controlled according to the standards of crossover trials. We tested whether the intervention–period interaction could be removed from the model.

The analysis of the secondary outcomes followed the same procedure as described above, with the addition of baseline characteristics as covariates to control for bias and confounding. In cases of missing data or incomplete data records, no imputation was performed, and an intention-to-treat analysis was used.^11^

### Prediction analysis

To further investigate whether a special set or combination of baseline characteristics may determine if a patient will respond to oxygen therapy or not, a *post hoc* analysis was performed. A responder was defined by a reduction in sPAP ≥10 mmHg when breathing oxygen compared with placebo air. A logistic regression analysis was performed to determine how useful certain baseline values might be in predicting a responder. For continuous variables, the optimal cut-off for the identified variables was estimated using receiver operating characteristic (ROC) curves.

In all analyses, a 95% confidence interval (CI) that excluded the null effect was considered as evidence of statistical significance. Analyses were performed using R-Studio software version 2023.12.1+402.

## Results

Model assumptions of homogeneity and normality of the residuals and random effects were met, and there were neither carry-over nor period effects.

**Figure 1:**
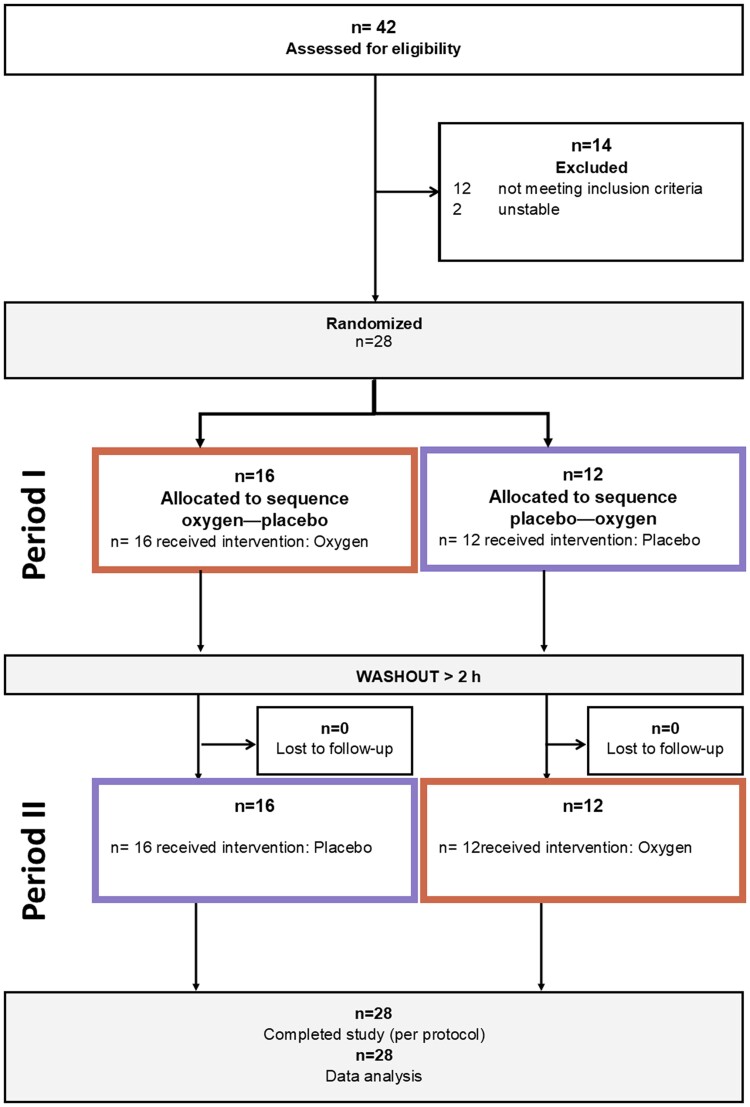
The study flow chart for crossover trials (adapted from CONSORT^12^). All participants who were randomized completed the study per protocol and were included into the analysis.

A total of 28 participants (26 PAH and 2 CTEPH, 24 females, age 45 ± 12 years) were included and completed the trial per protocol. According to the classical PAH phenotype, patients were predominantly female, were relatively young, and had severely impaired haemodynamics. The characteristics of the participants are listed in *Table 1*. The flow chart of the study is shown in *Figure 1*. All participants completed the study per protocol, and as the participants were predominantly younger to middle-aged females, the echocardiographic quality was good. A sensitivity analysis excluding the two patients with CTEPH was conducted to reduce the heterogeneity of the sample. Since the results were unchanged, we decided to report the data from all 28 patients.

**Table 1:** Baseline characteristics

	All	Responder	Non-responder
Number of participants	28	13 (46%)	15 (54%)
Female/male	24 (86%):4 (14%)	10 (77%):3 (23%)	14 (93):1 (7%)
Age (years)	45 ± 12	47 ± 14	43 ± 12
Pulmonary arterial hypertension	26 (93%)	13 (100%)	13 (87%)
Idiopathic	21 (75%)	12 (92%)	9 (60%)
Associated with:			
Congenital heart disease	4 (14%)	1 (8%)	3 (20%)
Connective tissue disease	1 (4%)	0(0%)	1 (7%)
Chronic thromboembolic pulmonary hypertension	2 (7%)	0 (0%)	2 (13%)
WHO functional class (I–IV)	II: 10 (36%), III: 18 (64%)	II: 3 (23%), III: 10 (77%)	II: 8 (53%), III: 7 (57%)
Body mass index (kg/m^2^)	25.6 ± 3.8	26.3 ± 3.9	25.1 ± 3.6
Current smoker/ex-smoker	0 (0%)/1 (4%)	0 (0%)/0 (0%)	0 (0%)/1 (7%)
SpO_2_ at rest (%)	87 ± 5	84 ± 6	89 ± 3
NT-proBNP (ng/L)	531 ± 844	404 ± 860	684 ± 908
Six minute walk distance (m)	499 ± 94	452 ± 104	470 ± 100
*Diagnostic right heart catheter data*			
Mean pulmonary artery pressure (mmHg)	52 ± 16	53 ± 14	52 ± 16
Pulmonary artery wedge pressure (mmHg)	11 ± 3	11 ± 3	10 ± 3
Pulmonary vascular resistance (WU)	16.3 ± 8.6	14.5 ± 9.2	17.7 ± 8.2
Cardiac output (L/min)	2.9 ± 0.9	2.7 ± 0.9	3.2 ± 1.0
*Arterial blood gases at time of diagnosis*			
Arterial partial pressure for oxygen (kPa)	8.5 ± 1.5	8.4 ± 1.2	8.6 ± 1.9
Arterial partial pressure for carbon dioxide ([kPa)	3.9 ± 0.5	4.0 ± 0.5	3.8 ± 0.6
Arterial oxygen saturation (%)	91 ± 5	91 ± 6	91 ± 5
*Echocardiography data*			
Left ventricular ejection fraction (%)	63 ± 5	63 ± 5	63 ± 5
Left atrial volume index (mL/m^2^)	23 ± 12	25 ± 12	20 ± 11
Right atrium area (cm^2^)	25.4 ± 12.4	26.7 ± 12.5	24.0 ± 12.7
Right atrium dilated	24 (86%)	10 (77%)	14 (93%)
Pericardial effusion	6 (21%)	3 (23%)	3 (20%)
Pulmonary hypertension*-targeted medication*			
Endothelin receptor antagonist	9 (32%)	6 (46%)	3 (20%)
Phosphodiesterase-5 inhibitor	28 (100%)	13 (100%)	15 (100%)
Calcium channel blocker	13 (46%)	6 (46%)	7 (47%)
Anticoagulation	19 (68%)	9 (69%)	10 (67%)
Diuretics	17(61%)	9 (69%)	8 (53%)
Nocturnal oxygen therapy	15 (54%)	7 (54%)	8 (53%)
24-h oxygen therapy	1 (4%)	1 (8%)	0 (0)

Data are presented as mean ± standard deviation, absolute numbers, or proportions.

WHO, World Health Organization; SpO_2_, oxygen saturation by pulse oximetry; NT-proBNP, N-terminal pro-B-type natriuretic peptide.

### Oxygen vs. placebo air

**Figure 2:**
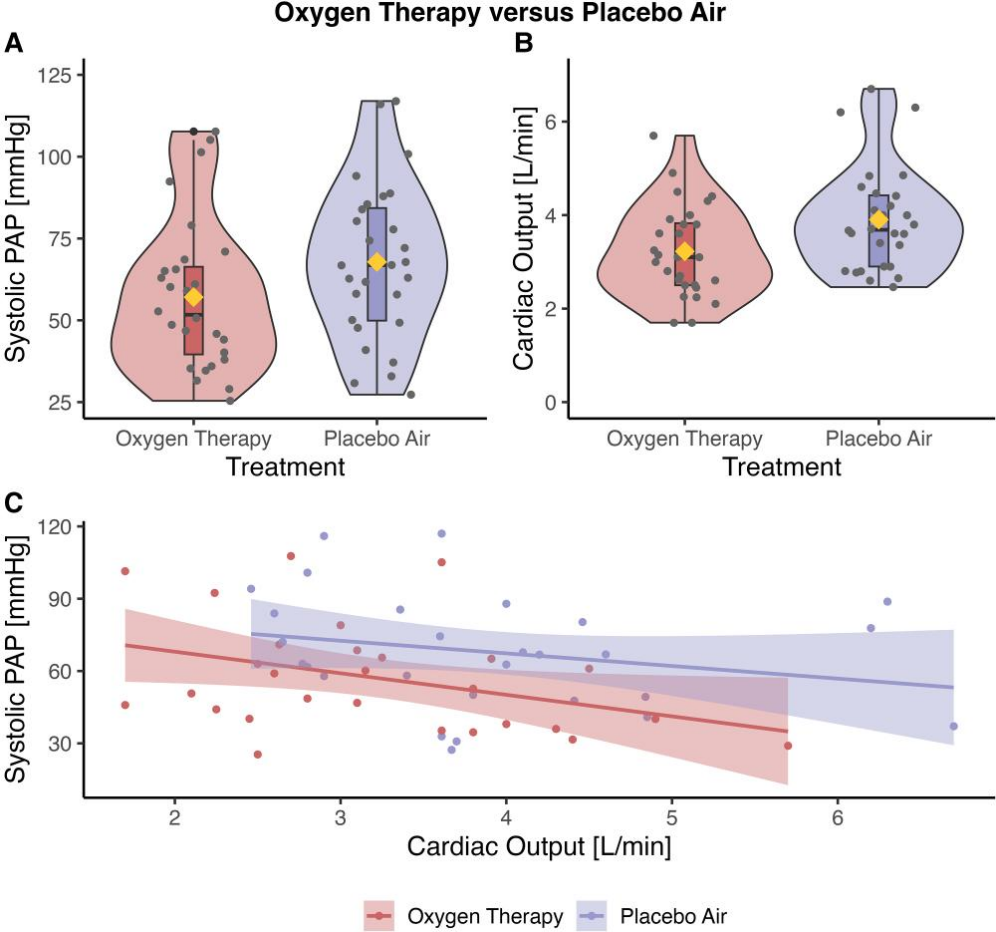
Echocardiographic parameters assessed after breathing 15 min of oxygen therapy vs. 15 min of placebo air. (*A*) The systolic pulmonary arterial pressure. (*B*) The cardiac output. The boxplots show the median, the inter-quartile ranges, the minimum, the maximum, and outliers. The violin plots represent the distribution of the data, while the width represents the frequency. The mean is displayed as diamonds, and the dots represent the individual data. (*C*) The pulmonary resistance. The individual patient data are represented by dots, and the lines show the regression lines with standard errors. There is a left shift with oxygen therapy in the lower left corner compared with placebo air, but the resistance remains unchanged as both systolic pulmonary arterial pressure and cardiac output decreased with oxygen therapy. The cardiac output decreases by a reduction in heart rate, while the stroke volume remains constant.

After 15 min of breathing oxygen, the sPAP was −11 mmHg (95% CI: −15 to −6 mmHg, *P* < 0.001) lower than after breathing 15 min of placebo air (*Figure 2*). Individual changes in sPAP are shown in Supplementary material online, *Figure S1*.

Heart rate and consequently CO were lower by 9 b.p.m. (95% CI: −14 to −5 b.p.m., *P* < 0.001) and −0.7 L/min (95% CI: −0.9 to −0.5 L/min, *P* < 0.001) with oxygen. After breathing 15 min of oxygen compared with placebo air, tricuspid annular plane systolic excursion (TAPSE)/sPAP was higher by 0.05 mm/mmHg (95% CI: 0.01–0.09 mm/mmHg, *P* = 0.024). None of the other echocardiographic parameters differed statistically significantly between oxygen and placebo air. The shunt fraction with oxygen (*Q*_s_/*Q*_t_) was 14 ± 4%. All outcomes are presented in Table 2.

**Table 2:** Echocardiographic data placebo air vs. oxygen therapy in Quito at 2850 m (PiO_2_ ≈ 60% of sea level). Values in bold indicate statistical significance.

Parameter	Placebo air (FiO_2_ ≈ 0.21)	Oxygen (FiO_2_ ≈ 0.95)	Mean difference (95% CI)	*P*-value
Tricuspid regurgitation pressure gradient (mmHg)	62 ± 22	52 ± 23	−10 (−14 to −5)	**<0.001**
Right atrial pressure (mmHg)	6 ± 4	5 ± 4	−1 (−1.6 to 0.3)	0.212
Systolic pulmonary arterial pressure (mmHg)	68 ± 24	57 ± 23	−11 (−15 to −6)	**<0.001**
Heart rate (beats/min)	74 ± 14	65 ± 10	−9 (−14 to −5)	**<0.001**
Velocity time integral (cm)	20.5 ± 4.7	20.3 ± 5.4	−0.2 (−1.5 to 1.6)	0.888
Stroke volume ((mL)	53 ± 13	53 ± 17	0 (−6 to 5)	0.929
Cardiac output (L/min)	3.9 ± 1.1	3.2 ± 1.0	−0.7 (−0.9 to −0.5)	**<0.001**
sPAP/CO (mmHg/L/min)	19 ± 10	20 ± 12	1 (−1 to 3)	0.380
Tissue Doppler index *S*′	10.8 ± 2.2	10.6 ± 2.3	−0.2 (−0.9 to 0.5)	0.575
TAPSE (mm)	15.9 ± 4.1	15.9 ± 3.9	0 (−0.8 to 0.9)	0.912
TAPSE/sPAP (mm/mmHg)	0.28 ± 0.16	0.33 ± 0.17	0.05 (0.01–0.09)	**0.024**
Fractional area change (%)	33 ± 7	32 ± 6	−1 (−3 to 2)	0.661
Right atrium area (cm^2^)	25.4 ± 12.4	24.5 ± 11.0	−0.9 (−2.6 to 1.4)	0.559
Right ventricle/left ventricle	1.4 ± 0.6	1.3 ± 0.4	−0.1 (−0.3 to 0.1)	0.232
Systolic blood pressure (mmHg)	108 ± 13	111 ± 12	3 (−3 to 6)	0.493
Diastolic blood pressure (mmHg)	66 ± 14	64 ± 13	−2 (−7 to 1)	0.076
Pulse oximetry oxygen saturation (%)	87 ± 5	97 ± 3	10 (9–12)	**<0.001**
Arterial partial pressure for oxygen (kPa)	7.6 ± 1.5	22.5 ± 9.7	14.9 (11.4–18.4)	**<0.001**
Arterial partial pressure for carbon dioxide (kPA)	4.0 ± 0.5	4.1 ± 0.5	0.1 (−0.2 to 0.0)	0.132
Shunt fraction (*Q*_s_/*Q*_t_) (%)	NA	14 ± 4	NA	NA
Arterial partial pressure for oxygen (kPa)	ΔPaO_2_ in responder 13.9 ± 10.8	ΔPaO_2_ in non-responder 16.3 ± 7.7	2.4 (−5.4 to 10.2)	0.530

Data are presented as mean ± standard deviation.

TAPSE, tricuspid annular plane systolic excursion; CO, cardiac output; sPAP, systolic pulmonary arterial pressure.

### Responders to high-dose oxygen therapy

**Figure 3:**
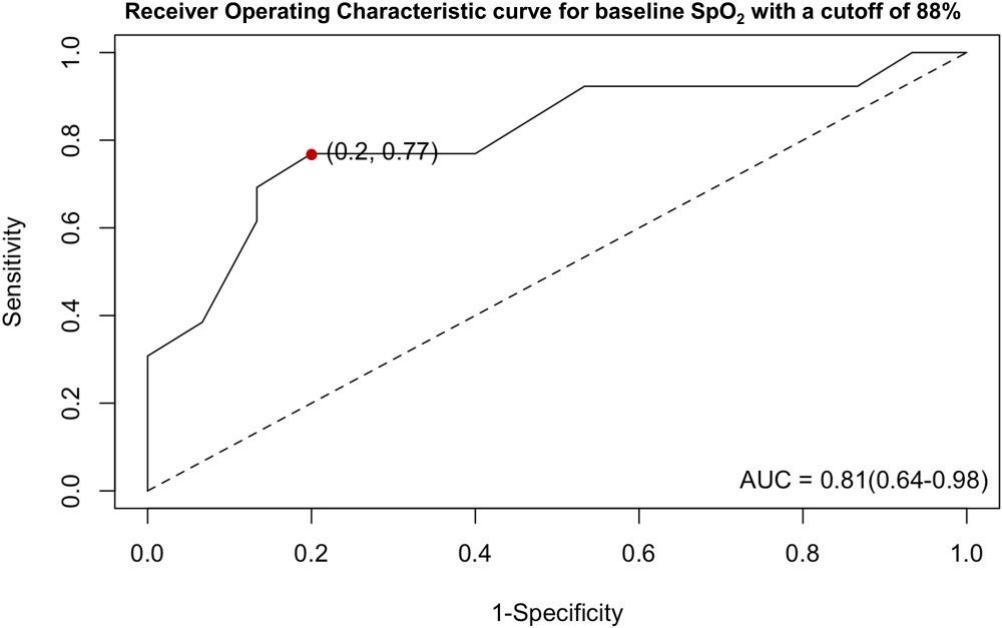
The receiver operating characteristic curve for baseline SpO_2_ to predict whether a patient will be a responder to oxygen therapy. The optimal cut-off was identified at 88%, with a sensitivity of 77% and a specificity of 80% (dot). AUC, area under the curve.

A total of 13 participants were identified as responders (sPAP reduction by ≥10 mmHg with oxygen). Logistic regression analysis showed that neither baseline RHC nor echocardiographic data or PH-targeted medication were useful in predicting whether a patient might be a responder to oxygen therapy. The best predictor was baseline SpO_2_, with a cut-off point of 88% identified by ROC curve analysis [area under the curve (AUC) = 0.81, sensitivity = 0.77, specificity = 0.80, see *Figure 3*]. The odds ratio of being a responder was 21.7 (95% CI: 3.0–155.4, *P* = 0.002) for patients with a baseline SpO_2_ ≤ 88% compared with those with a baseline SpO_2_ > 88%.

## Discussion

In this randomized, single-blind, placebo-controlled crossover trial, we found that high-dose oxygen therapy significantly reduced sPAP compared with placebo air, in 28 patients with PVD permanently residing at an altitude of 2850 m. Furthermore, the CO was lower with oxygen therapy, mainly due to a reduction in heart rate, but pulmonary resistance was unchanged. TAPSE/sPAP was significantly higher under oxygen, indicating improved coupling between the right ventricle and the pulmonary arteries. Forty-six per cent of the patients were responders to oxygen therapy, defined as a decrease in sPAP by ≥10 mmHg with oxygen therapy, and it was found that SpO_2_ on ambient air at 2850 m using a cut-off of 88% is useful in predicting whether a patient will be a responder.

The severity of PVD at baseline and the reduction in PAP with PH-targeted medication are of prognostic importance for survival in patients with PVD living at low altitude.^13,14^ In a previous study, it was shown that hyperoxia (FiO_2_, 1.0) applied at 470 m of altitude via a facial mask reduced sPAP from 54 to 48 mmHg (11%) in patients with PVD.^15^ Therefore, the magnitude of sPAP reduction with oxygen observed in this trial (from 68 to 57 mmHg, 16%) in patients with PVD permanently living at 2850 m of altitude was higher.

Elevated sPAP represents right ventricular overload, which impairs right ventricular function.^1^ However, not solely sPAP was lower with oxygen. TAPSE/sPAP as an indicator of right ventricular–arterial coupling and thus right ventricular contractile response to increased afterload was higher with oxygen compared with placebo air.^16,17^ This suggests that oxygen improved right ventricular contractile function compared with placebo air.

Of interest, CO was also lower with oxygen compared with placebo air, leading to an unchanged total pulmonary resistance. Lower CO with oxygen can be explained by a 25% increase in systemic vascular resistance.^18^ The reduction in CO was driven by a decrease in heart rate of 9 b.p.m. with oxygen. Since stroke volume was unchanged, the decrease in heart rate might be a sign of less sympathetic over excitation.^19^ This was also observed in PVD at low altitude, healthy volunteers, and coronary artery disease, while in patients with heart failure CO decreased during oxygen therapy through a reduction in stroke voloume.^18^ The 12% decrease in heart rate, with preserved stroke volume, indicates that right ventricular load is reduced with oxygen therapy, which is an important observation for patients with PVD. Similar heart rate reduction was also found when patients with PVD exercised with high-dose oxygen vs. placebo.^19^ Recently, sotatercept, which is a novel pharmacological treatment for PAH, has been shown in a Phase-3 study to improve the 6-min walk distance, time to clinical worsening, and haemodynamics in patients with PAH.^20^ Interestingly, Sotatercept also improved haemodynamics by reducing PAP, while CO was unchanged.^21^

When total pulmonary resistance remains unchanged while sPAP declines during oxygen therapy, the lower CO must be considered as main factor to the decrease in pressure. When the oxygen content of the arterial blood is increased, as was here the case with oxygen therapy, even with lower CO, the oxygen delivery remains similar. In other words, a decrease in CO even in patients with PVD should not be problematic as long as the oxygen delivery remains similar due to the increased blood oxygen content.^18^ A lower CO due to the lower HR with unchanged SV might even be considered as relieve for the cardiopulmonary system. The opposite physiological reaction can be observed when patients with PVD are acutely breathing hypoxic air, which leads to an increased PAP and increased CO.^22^

Vasoreactivity testing during RHC is not yet available in Ecuador. Although oxygen is a vasodilator in the pulmonary vessels, it cannot replace nitric oxides for vasoreactivity testing. Nevertheless, for a positive vasoreactivity test, a decrease in mPAP of ≥10 mmHg, to below 40 mmHg with constant CO, must be achieved, which is not approximately the case here.^1,23^ In addition, as the availability of PAH-targeted medication in Ecuador is still limited (see *Table 1*), around 50% of the patients with PVD included in this study empirically received calcium channel blockers. However, considering the severely compromised haemodynamics in the present PVD cohort with a mean sPAP of 68 mmHg with therapy, the vast majority of the presently investigated patients with PVD are very unlikely to be vasoreactive.

We identified 13 (46%) responders to oxygen therapy defined by a decrease in sPAP ≥10 mmHg. None of the demographic, RHC, or echocardiographic data at baseline differed between responders and non-responders. Furthermore, neither medical therapy nor nocturnal oxygen therapy did influence the responsiveness to oxygen therapy. Only SpO_2_ while breathing ambient air at 2850 m was shown to be a useful predictor for sPAP responsiveness to oxygen. An SpO_2_ of 88% with ambient air was identified as an optimal cut-off for the highest diagnostic accuracy to identify a responder (*Figure 3*). When a patient had a baseline SpO_2_ of <88%, the chance to be a responder to oxygen therapy was around 22 times higher compared with patients with a baseline SpO_2_ ≥ 88%, in 95% of the times. With an AUC of 0.81, SpO_2_ represents a quite accurate, simple, and cheap test that might help decision-making, especially in countries with limited medical infrastructure. However, whether improving haemodynamics by supplemental oxygen in these patients with PVD would translate into improved patient-reported outcomes, quality of life, exercise capacity, and survival remains to be investigated. In addition, the oxygen dose of 10 L/min was high, so it is unknown whether a lower dose, as usually given in every day practice, would have similar effects.

The increase in PaO_2_ (ΔPaO_2_) with oxygen was similar between responders and non-responders. Furthermore, the calculated shunt fraction (*Q*_s_/*Q*_t_) with 100% oxygen was 14%, which is identical to that found in patients with PVD at 470 m of altitude.^15^ Thus, undetected cardiac or pulmonary shunts that might confound the responsiveness to oxygen therapy are unlikely.

Recent European guidelines recommend oxygen therapy only for hypoxaemic patients with PVD at rest.^1^ In the cohort of patients with PVD who live at high altitude, we found that the haemodynamic response to oxygen is dependent on the SpO_2_ with ambient air.

High-dose oxygen therapy was previously shown to increase exercise capacity in patients with PVD.^8,19^ However, whether lower doses of oxygen as applied via nasal cannula in everyday practice with up to 4 L/min would be similarly beneficial to improve exercise performance remains to be elucidated. Nocturnal or domiciliary oxygen therapy, which is comparatively easy to apply, has been shown to improve daytime performance even when patients are breathing ambient air during the day.^7,9^ Nevertheless, long-term effects or potential benefits for patients with PVD have not been investigated so far.

### Limitations

The dose of oxygen chosen for this study on haemodynamics (10 L/min) via rebreathing facial mask was high, and thus, we do not know the haemodynamic effects of lower oxygen doses as used at home, during nights, or ambulatory in hypoxaemic patients with PVD. There were 15 patients on nocturnal and one patient on 24-h oxygen therapy, which might have influenced the chemosensitivity to high-dose oxygen therapy. However, oxygen therapy at baseline had no statistically significant effect on the responsiveness to oxygen therapy within the logistic regression analysis. The sample size of our trial was rather small; however, in a crossover trial, it is possible to reduce the sample size by 50–75% without losing power. Therefore, the sample size of 28 patients was considerable, given the relatively rare collective with predominantly typical phenotype of patients with PVD, and we did not have a carry-over or period effect. This phenotype is characterized by low age, female predominance, and severe haemodynamic compromise, all of whom live at high altitude. Whether these results are applicable to different populations living at varying altitudes is uncertain and requires further investigation. The investigators were not blinded to the study intervention.

## Conclusions

High-dose oxygen therapy in prevalent patients with PVD, predominantly PAH, who permanently live at high altitude near 2850 m, significantly lowered sPAP and reduced CO, resulting in an unchanged pulmonary resistance. This indicates oxygen-induced pulmonary vasodilatation, and along with the improved right ventricular–arterial coupling, it suggests a reduced load on the right heart with supplemental oxygen. Forty-six per cent of patients responded to oxygen therapy (sPAP at least −10 mmHg), and a SpO_2_ cut-off of 88% was an accurate, simple, and inexpensive test to predict responders to high-dose oxygen therapy. Whether longer-term oxygen therapy given to patients with PVD living at higher altitude would decrease pulmonary vascular resistance remains to be studied.

## Supplementary Material

oeae097_Supplementary_Data

## Data Availability

The data from this study will be available from the corresponding author upon reasonable request.
