# Supplementary figures and images for: The acute effect of high-dose supplemental oxygen on haemodynamics assessed by echocardiography in patients with pulmonary vascular disease living in Quito at 2850 m: a randomized, single-blind, placebo-controlled crossover trial
Source: Eur Heart J Open. 2024 Dec 2;4(6):oeae097. doi: 10.1093/ehjopen/oeae097 (PMC11653896; doi:10.1093/ehjopen/oeae097)

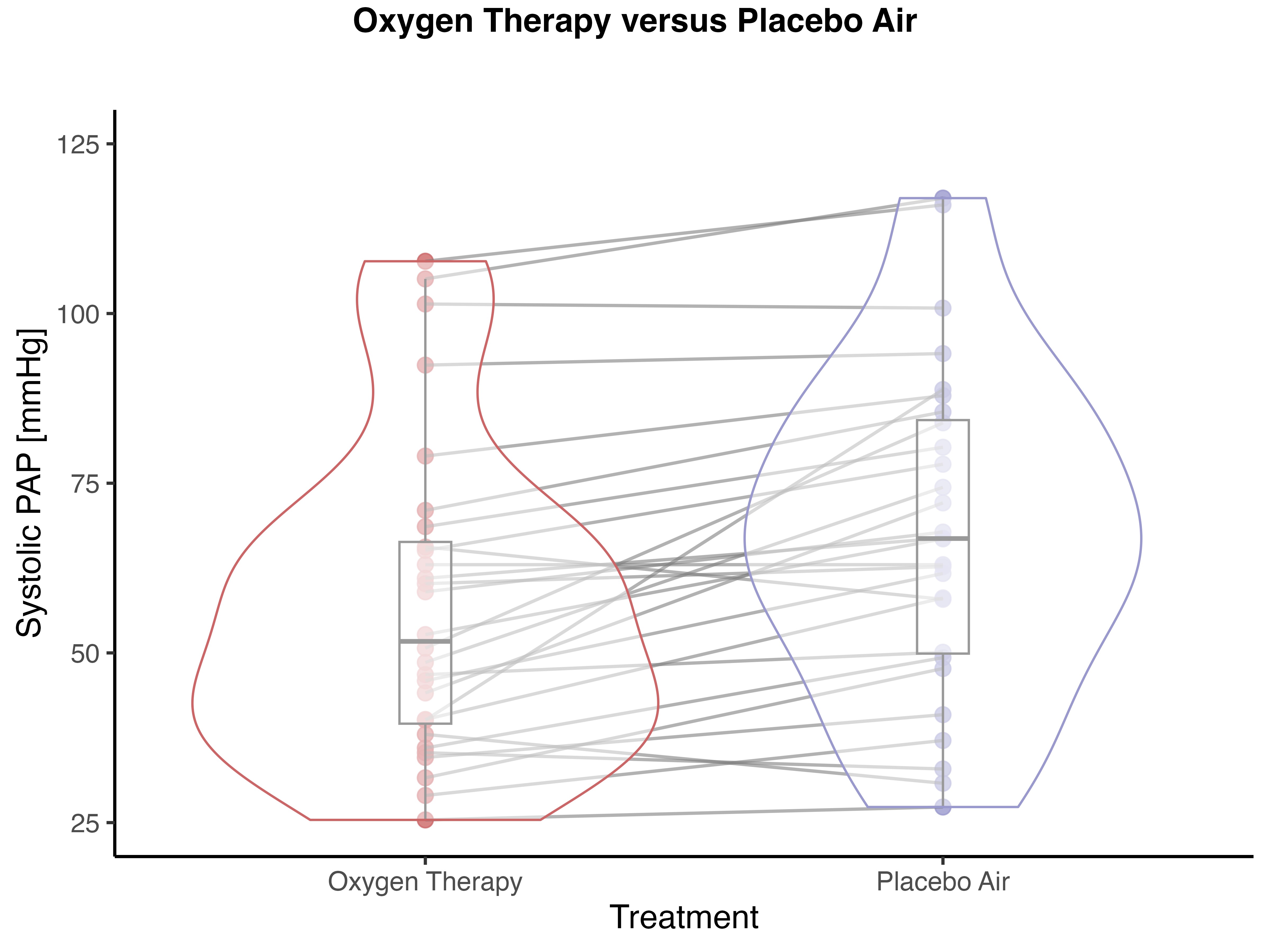

Supplement: oeae097_Supplementary_Data [file oeae097_supplementary_data.jpeg]
